# Identification of Circular RNAs Regulating Islet *β*-Cell Autophagy in Type 2 Diabetes Mellitus

**DOI:** 10.1155/2019/4128315

**Published:** 2019-11-07

**Authors:** Chao Bai, Wenwen Yang, Yao Lu, Wei Wei, Zongbao Li, Li Zhang

**Affiliations:** ^1^Department of Vascular Thyroid Surgery, First Hospital Affiliated to Xinjiang Medical University, Urumqi 830054, China; ^2^Department of Endocrinology, Second Hospital Affiliated to Xinjiang Medical University, Urumqi 830063, China; ^3^Department of Endocrinology, Sanya Central Hospital, Sanya 572000, China

## Abstract

This study is to identify the circular RNA (circRNA) expression profile that is functionally related to pancreatic islet *β*-cell autophagy and their potential regulation mechanisms in type 2 diabetes mellitus (T2DM). T2DM rat model was constructed by administration of high-fat and high-sugar diet. *β*-cells were isolated from islets by flow cytometry. CircRNA expression profile in *β*-cells was detected by circRNA microarrays, and the differentially expressed circRNAs were identified and validated by qRT-PCR. MicroRNA (miRNA) target prediction software and multiple bioinformatic approaches were used to construct a map of circRNA-miRNA interactions for the differentially expressed circRNAs. A total of 825 differentially expressed circular transcripts were identified in T2DM rats compared with control rats, among which 388 were upregulated and 437 were downregulated. Ten circRNAs were identified to have significant differences by qRT-PCR. GO analysis enriched terms such as organelle membrane and protein binding and the top enriched pathways for the circRNAs included MAPK signaling pathway. The differentially expressed circRNAs might involve in MAPK signaling pathway, apoptosis, and Ras signaling pathway. We speculate that these circRNAs, especially rno_circRNA_008565, can regulate the autophagy of islet *β*-cells via interactions with miRNA. Dysregulation of several circRNAs may play a role in T2DM development, and rno_circRNA_008565 may be a potential regulator of *β*-cell autophagy.

## 1. Introduction

Diabetes is one of the chronic noninfectious diseases that threaten human health worldwide. Because of the aging population, the prevalence of bad lifestyle, and the change of dietary structure, type 2 diabetes mellitus (T2DM) shows a rapid increase in China. The prevalence rate of adult diabetes in China was 5.49% in 2000-2001 [1], which has been increased to 11.6% in 2010 [2]. The latest research shows that the incidence of T2DM in adults is as high as 11.6% [2], which is expected to increase [3]. T2DM patients suffer from the disease for long term, which puts a heavy burden on individuals, families, and society [4].

Decreased number of islet *β*-cells and their dysfunction are one of the important pathogeneses of T2DM. Autophagy is one of the self-protection mechanisms of islet *β*-cells [5]. The substrate to be degraded is encapsulated into autophagosomes by a bilayer membrane structure under the control of autophagy-related genes (ATGs) and then transported to lysosomes for membrane fusion. In the lysosomes, a series of hydrolases digest the cell's own proteins or organelles to support islet *β*-cell metabolism and organelle renewal [6, 7]. Under physiological conditions, autophagy in islet *β*-cells is maintained at a lower level to keep the recycling of cytoplasm, organelles, and proteins, which protects cells and promotes cell survival. However, defective or excessive autophagy can result in the apoptosis of islet *β*-cells. For example, after knocking out ATG7 in mouse islet *β*-cells, the mice showed impaired glucose tolerance, decreased serum insulin levels, and increased apoptosis while decreased proliferation of islet *β*-cells [8].

It is reported that autophagy levels in *β*-cells are positively correlated with insulin levels, suggesting that autophagy may be involved in the process of insulin storage and release [9]. Mice with ATG7-specific knockout in islet *β*-cells showed hypoinsulinemia and hyperglycemia [10]. ATG7 knockout mice not only have significantly reduced number of islet *β*-cells [11] but also show a marked decrease in islet *β*-cell function, which is characterized by significantly lower basal insulin secretion and high glucose-induced insulin secretion [12]. Therefore, autophagy of islet *β*-cells is essential for maintaining islet *β*-cell function, while autophagy abnormality can lead to decreased *β*-cell number and abnormal *β*-cell function.

Circular RNA (circRNA) is a newly identified type of noncoding RNAs that are mainly composed of more than one exon and widely expressed in eukaryotic cells [13]. CircRNA is highly conserved and has tissue, time, and disease specificity [14, 15]. The circRNAs are enriched in miRNA response elements and thus can act as miRNA sponges in cells [16–19]. In general, they act as competitive endogenous RNAs that bind to certain miRNAs, thereby releasing the inhibition of miRNA on their target genes and upregulating the expression level of the target genes [20]. Therefore, circRNA may regulate the development of T2DM through targeting certain miRNAs.

Studies [21, 22] have reported that a variety of differentially expressed genes and related signaling pathways are involved in islet *β*-cell autophagy of T2DM. However, whether circRNAs are involved in T2DM by regulating the autophagy of islet *β*-cells is still unclear. Herein, we sort to identify the circRNA expression profile that is functionally related to pancreatic islet *β*-cell autophagy and their potential regulation mechanisms in T2DM. Our study will help understand the pathogenesis of T2DM and may provide new strategy for the treatment of T2DM.

## 2. Materials and Methods

### 2.1. Animals

A total of 22 six-week-old male Sprague Dawley (SD) rats were purchased from Cavens Experimental Animals Co., Ltd. (Changzhou, China; Permission No. 201608927). The rats were raised in separated IVC cages with room temperature at 26°C, 12 h shift light cycle. The experimental protocol was ethically approved by Second Hospital Affiliated to Xinjiang Medical University.

### 2.2. T2DM Model Establishment

The rats were randomly divided into 2 groups (*n* = 11 each): the control group and the T2DM model group. The T2DM group of rats was administered a high-fat and high-sugar diet (10% lard, 20% sucrose, 2.5% cholesterol, 1% cholic acid salt, and 66.5% conventional feed) for 12 weeks to induce insulin resistance. Then, T2DM rats were fasted for 12 h but with free access to water. After this, rats received intraperitoneal injection of 35 mg/kg streptozotocin dissolved in sodium citrate buffer (0.1 mol/L, pH 4.5). To determine successful establishment of the DM model, blood glucose levels were measured 48 h later using Roche ACCU-CHEK (Roche, Germany). Rats with blood glucose >16.7 mmol/L were considered as success T2DM model establishment. Finally, T2DM was successfully established in all 11 rats and these rats were used in downstream analysis. The control group of rats was fed with regular diet and received intraperitoneal injection of sodium citrate buffer (0.1 mol/L, pH 4.5).

### 2.3. Isolation of Rat Islet *β*-Cells

Immediately after T2DM model establishment, animals were euthanized. Collagenase (Cat#: Sigma C9263; Sigma) is injected into the common bile duct of euthanized animals. The pancreas is then excised and digested at 37°C for 30 min with vigorous shaking. All the digested fractions are then collected. The islet cells were washed with Hanks (without Mg and Ca) and cultured for 4 min under continuous shaking at 37°C followed by an additional shaking at 37°C for 3 min in 1640 medium (Hyclone). Then, islet *β*-cells were sorted by flow cytometry (BD-Aria II).

### 2.4. CircRNA Microarray Analysis

For circRNA profiling, RNA from rat islets was extracted from islet *β*-cells with TRIzol reagent (Thermo Fisher, USA). Total RNA was analyzed by 1.5% agarose PAGE and NanoDrop ND-100 (Thermo Fisher, USA). CircRNA expression profiling and data analysis were carried after out using circRNA Array (Arraystar). Scanned images (in tiff format) by Agilent Scanner G2505C were imported into Agilent Feature Extraction software (version 11.0.1.1) for raw data extraction. Raw data were normalized with quantile algorithm, LIMMA package in R. CircRNAs that were differentially expressed between the two groups were estimated by Agilent GeneSpring GX with fold-change filtering and Student's *t*-tests. CircRNAs with fold changes >2.0 and *P* values ≤0.5 were defined as significantly differentially expressed.

### 2.5. Real-Time PCR Validation of CircRNAs

RNA was reverse-transcribed into cDNA using AMV Reverse Transcriptase (Thermo #K1622). Quantitative real-time PCR of circRNAs was performed using SYBRGreen PCR Kits (Thermo F-415XL) according to the manufacture's construction. For primer design, the circRNA sequences were obtained through circRNA database (http://www.circbase.org/). Then, sequences upstream and downstream of backsplice were combined. Finally, primers were designed for this 300 bp sequence using Primer 5. The expression of small nuclear *β*-actin was used as internal control. The reaction mixture was incubated for 1 cycle at 95°C for 10 min, followed by 40 cycles at 94°C for 20 s, 55°C for 20 s, and 72°C for 20 s, and 1 cycle at 72°C for 1 min. Primers used for amplification are shown in Table 1. The relative expression levels were evaluated using the 2^−ΔΔCt^ method.

### 2.6. Analysis of the Related Messenger RNAs of Differentially Expressed CircRNAs

According to the position information of the differentially expressed circRNAs in islet *β*-cells of T2DM model group and the control group, the protein-coding genes associated with the genomic position of the circRNA were obtained. The functional roles of the circRNA-related genes were further analyzed using Gene Ontology (GO; geneontology.org), and the biological pathways of circRNA target genes were identified using Kyoto Encyclopedia of Genes and Genomes (KEGG; http://www.kegg.jp). The GO and KEGG analyses were performed using Bayesian network, Genes-Networks, and GO analysis software.

### 2.7. Prediction of CircRNA/MicroRNA Interactions

The circRNA/microRNA interaction was predicted using Arraystar's miRNA target prediction software based on miRanda and TargetScan. To establish circRNA-miRNA network, we searched MREs on the top 10 differentially expressed circRNAs using the software and then selected the miRNAs according to seed-matching sequences.

### 2.8. Statistical Analysis

Statistical analysis was conducted using the SPSS Statistics 18.0 software (IBM Corp, New York, NY). Significant difference between control and T2DM groups was compared using Student's *t* test. *P* < 0.05 was considered as statistically significant.

## 3. Results

### 3.1. Microarray Analysis of CircRNAs in Islet *β*-Cells

**Figure 1:**
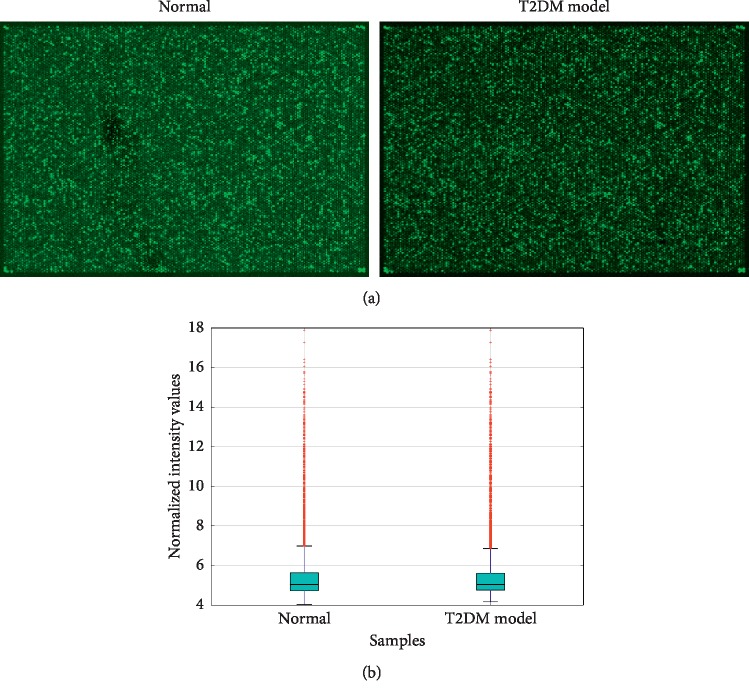
CircRNA microarray expression data between *β*-cells from normal and T2DM rat islets. (a) The typical results of circRNA microarray from control and T2DM rat islet *β*-cells. Each bright spot represents a circRNA. (b) Normalized data were plotted as a box plot to determine the overall characteristics of distribution.

High-throughput microarray was used to determine circRNA expression in islet *β*-cells from control and T2DM groups of rats. Representative microarray results are shown in Figure 1(a). Raw data of the chip were normalized with quantile algorithm to obtain normalized intensity, which was then transformed into log2 values. The box plot (Figure 1(b)) was used to display the overall characteristics of sample data distribution, which showed that the distributions of data from two groups were almost the same after normalization.

### 3.2. Identification of Differentially Expressed CircRNAs

**Figure 2:**
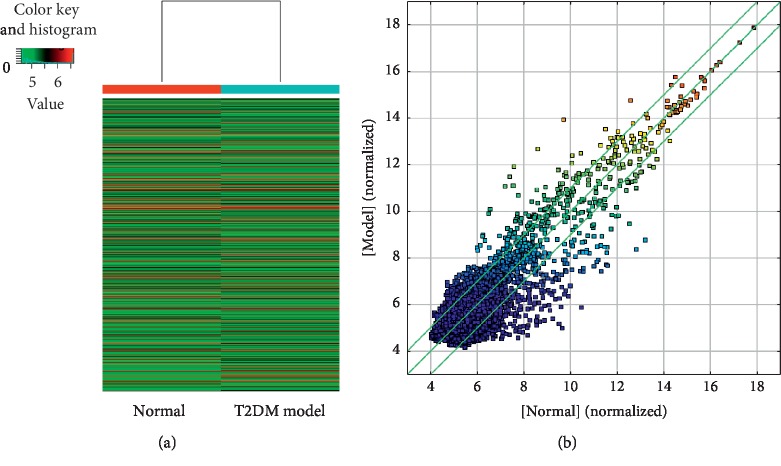
Identification of differentially expressed circRNAs between *β*-cells from normal and T2DM rat islets. (a) The heatmap depicts an intuitive graphical representation of gene expression in different samples after hierarchical clustering of all circRNAs. (b) Scatter plots of chip data assessing overall distribution of the two sets of data. The *X* and *Y* axis values in the scatter plot are the normalized signal values of the samples (log2 scaled). Portions beyond the lines indicate differentially expressed circRNA (fold change > 2.0 and *P* values ≤ 0.5).

Heatmap and scatter plots showed the variations in circRNA expression between T2DM model group and control group. A total of 8117 circRNAs were detected by the circRNA microarray (Figure 2(a)). We identified 825 circRNAs that were differentially expressed between the control and T2DM rats. Three hundred and eighty-eight of these circRNAs were upregulated, and 437 were downregulated in T2DM rats (Figure 2(b)). Some differentially expressed circRNAs are shown in Table 2.

### 3.3. Real-Time PCR Validation of Differentially Expressed CircRNAs

**Figure 3:**
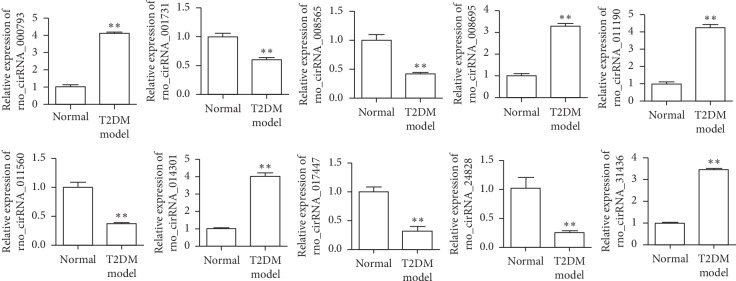
Expression of circRNAs (rno_circRNA_011190, rno_circRNA_014301, rno_circRNA_000793, mmu_circRNA_31436, rno_circRNA_008695, rno_circRNA_001731, rno_circRNA_017447, rno_circRNA_008565, rno_circRNA_011560, and mmu_circRNA_24828) detected via real-time PCR using islet *β*-cells from 8 control and 8 T2DM rats. ^*∗∗*^*P* < 0.01, Student's *t* test.

To verify microarray expression data, expression of 10 circRNAs (circRNA_011190, circRNA_014301, circRNA_000793, circRNA_31436, circRNA_008695, circRNA_001731, circRNA_017447, circRNA_008565, circRNA_011560, and circRNA_24828) was detected via real-time PCR in *β*-cells from additional 8 control and 8 T2DM rats (Figure 3). These 10 circRNAs were selected based on their most significantly differential expression between control and T2DM groups in microarray data. These data demonstrated that the results of microarray can be validated by qRT-PCR.

### 3.4. GO Enrichment Analysis of the Validated 10 CircRNAs

**Figure 4:**
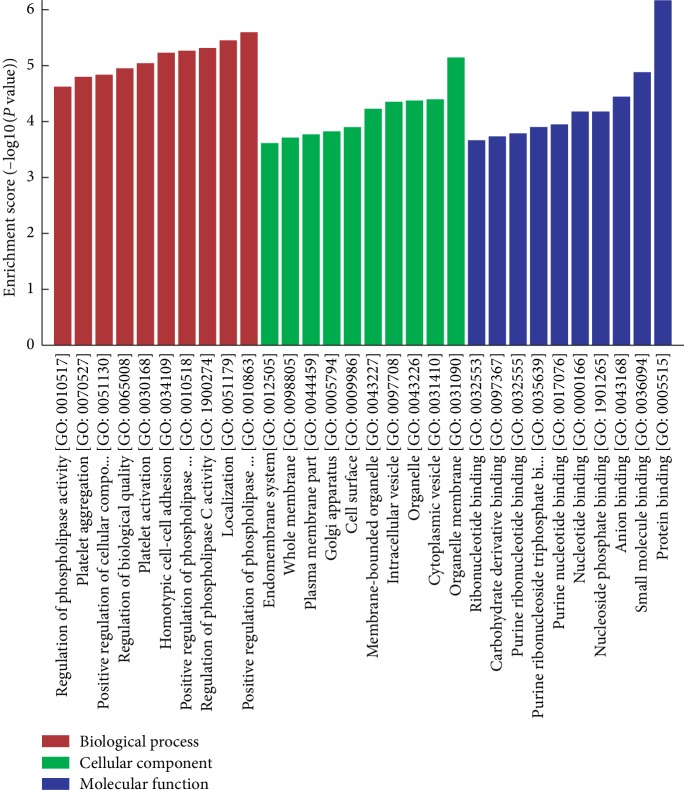
GO analysis of the target genes of circRNAs. The target genes of the 10 validated circRNAs were identified using bioinformatics tools. Functions of the target genes of the circRNAs were enriched by GO analysis.

To determine the relationship between differentially expressed circRNAs and T2DM development, GO enrichment analysis was performed. The top 30 enriched GO terms for the validated 10 differentially expressed circRNAs were identified (Figure 4). The circRNAs were significantly associated with the biological processes (BP) associated with the regulation of the metabolism of four phospholipases. In addition, 187 genes were associated with localization in BP. The most enriched GO term in cellular component (CC) was organelle membrane, while the most enriched GO term in molecular function (MF) was protein binding. There were 6 GO terms related to nucleic acid.

### 3.5. KEGG Analyses for CircRNA-Related Genes

**Figure 5:**
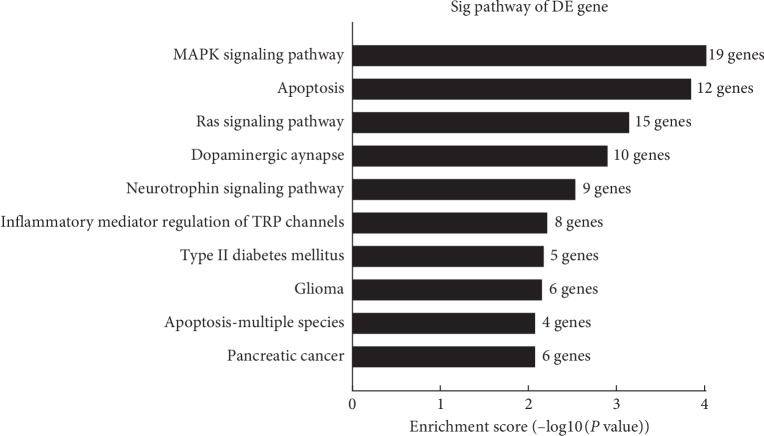
KEGG pathway analysis of the circRNA-related genes. The top 10 pathways of the related genes of differentially expressed circRNAs were identified using KEGG analysis according to the number of enriched genes.

The top 10 KEGG pathways were also identified for the differentially expressed circRNAs (Figure 5). The top enriched pathways for the circRNAs included MAPK signaling pathway (19 genes), apoptosis (12 genes), and Ras signaling pathway (15 genes). Importantly, 5 genes were enriched in type 2 diabetes mellitus pathway.

### 3.6. Construction of the CircRNA/MicroRNA Interaction Network

**Figure 6:**
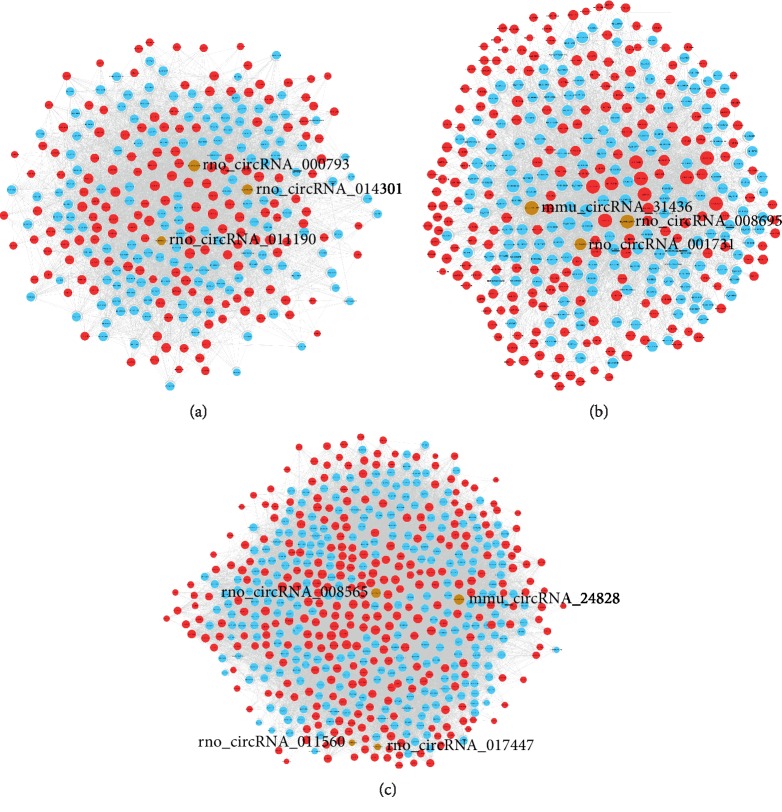
The circRNA/miRNA network analysis. (a) The network consists of rno_circRNA_000793, rno_circRNA_011190, and rno_circRNA_014301 (brown modes), mRNAs (blue nodes), and their target miRNAs (red nodes). (b) The network consists of mmu_circRNA_31436, rno_circRNA_001731, and rno_circRNA_008695 (brown modes), mRNAs (blue nodes), and their target miRNAs (red nodes). (c) The network consists of mmu_circRNA_24828, rno_circRNA_008565, rno_circRNA_011560, and rno_circRNA_017447 (brown modes), mRNAs (blue nodes), and their target miRNAs (red nodes).

To determine the function of circRNAs, interactions between circRNAs and their target miRNAs were theoretically predicted by conserved seed-matching sequence using the Arraystar software. The 10 validated differentially expressed circRNAs were predicted according to the complementary miRNA-matching sequence. A total of 1286 miRNAs could be combined with circRNAs (Figure 6). The three networks constructed with rno_circRNA_000793, rno_circRNA_011190, and rno_circRNA_014301 (brown nodes, Figure 6(a)), mmu_circRNA_3143, rno_circRNA_001731, and rno_circRNA_008695 (brown nodes, Figure 6(b)), and mmu_circRNA_2482, rno_circRNA_008565, rno_circRNA_011560, and rno_circRNA_017447 (brown nodes, Figure 6(c)) were depicted, respectively.

### 3.7. Identification of the CircRNAs Associated with Islet *β*-Cell Autophagy

In order to find the most critical circRNA for islet *β*-cell autophagy, 14 circRNA target genes related to autophagy were identified by integral analysis of GeneCards database and the results of circRNA target genes (mRNA) in the circRNA-miRNA-mRNA network. The results (Table 3) showed that rno_circRNA_008565 had the highest score relevant to autophagy.

## 4. Discussion

Autophagy is a highly conserved pathway in eukaryotic cells, a process in which abnormal proteins and organelles are transported to lysosomes for further degradation [23]. Autophagy plays an important role at the cellular level and is an important mechanism for cell self-protection. There are five steps in autophagy: autophagy induction, vesicle nucleation, expansion and completion, lysosome fusion (autophagolysosome formation), and breakdown (degradation and recycling) [24]. In eukaryotic cells, autophagy is important for biological processes such as metabolism of their own substances, removal of toxic substances, renewal of organelles, and maintenance of cell homeostasis [25]. Autophagy has different effects on cell survival since autophagy can eliminate harmful substances and maintain the stability of the body's environment [26]. However, excessive autophagy induction can lead to cell death [27].

CircRNAs are a novel type of universal and diverse endogenous noncoding RNA. The 3′ and 5′ ends of circRNAs can be joined together by covalent bonds; this leads to circularization, resistance to RNA exonuclease-mediated degradation, and high stability [13, 28]. CircRNA also has cell specificity, tissue specificity, and timing specificity. Compared with microRNAs, circRNA is a more ideal biomarker for diseases, providing new targets for the subsequent treatment of various diseases [29].

Many studies [30–33] have uncovered the role of circRNAs in multiple diseases. However, the function of circRNAs in diabetes is emerging. For example, a study shows that hsa_circRNA_0054633 is closely related to gestational diabetes (GDM) [34]. Hsa_circRNA_0054633 was elevated in serum in pregnant women and in the third trimester of pregnancy and was positively correlated with postprandial blood glucose and glycosylated hemoglobin. This circRNA further affected the epigenetic expression of placenta by participating in the regulation of maternal serum glucose metabolism. This study suggests that hsa_circRNA_0054633 has a highly diagnostic value for GDM.

A study on patients with T2DM and healthy control suggests that there are 489 differentially expressed circRNAs in the peripheral blood of T2DM patients compared with the healthy control, and the researchers showed that hsa-circ-0054633 is involved in mitosis cell cycle block and molecular metabolism [35]. Thus, hsa-circ-0054633 may be involved in the pathogenesis of diabetes by affecting cell metabolism and cell cycle, which may be used as a marker for the diagnosis of T2DM in peripheral blood.

Mitogen-activated protein kinase (MAPK) pathway plays a key role in gene expression regulation and cytoplasmic activities. The MAPK signaling pathway is a classical pathway regulating autophagy [36]. Our data suggest that circRNA may affect the occurrence and progression of diabetes by participating in the MAPK pathway. The MAPK signaling pathway can be activated by hyperglycemia and is involved in inflammatory responses and apoptosis [37], while inflammatory responses and abnormal apoptosis may be associated with diabetes.

CircRNA can regulate insulin signaling and can be used as biomarker for cell metabolism and T2DM [35, 38]. In a preliminary study by Ye et al., peripheral blood has-circ-000094 could be used as a diagnostic marker for T2DM. They found the elevated expression of has-circ-000094 in patients with T2DM inhibited the expression of P21 through repressing hsa-miR-370-5p, further leading to the deactivation of kinase PAK1 [39]. By searching the GeneCards database, we identified 14 circRNAs that may be related to autophagy. MAPK8 (JNK), one of the rno_circRNA_008565 target genes, had highest score with autophagy. miRNA binding prediction found that rno-miR-504 had most binding sites (4 sites) with rno_circRNA_008565 and had the most stable binding structure, suggesting that rno_circRNA_008685 may regulate MAPK8 (JNK) and the autophagy of rat islet *β*-cells by inhibiting rno-miR-504. However, the specific mechanism still needs further investigation.

C-Jun N-terminal protein kinase (JNK) is a member of the MAPK family, which is closely related to autophagy. Wei et al. [40] reported that the JNK pathway has critical role in the autophagy triggered by cell nutrition deficiency. It upregulates autophagy levels mainly through phosphorylation of Bcl2 family proteins, which further blocks these proteins to form complexes with beclin-1 [41–43]. Another study also showed that JNK could activate autophagy through directly interacting with Atg7 [44]. Therefore, JNK's regulation of autophagy is very complex, and its effect on cell fate depends on the environment and upstream signals in cells.

JNK signaling is an important pathway in regulating the autophagy of islet *β*-cells [45]. After transient stimulation by palmitic acid in rat pancreas INS-1 cells, the transformation of microtubule-associated protein LC3-I to LC3-II, a marker of autophagy activation in islet *β*-cells, is enhanced. This further induces the formation of classical autophagosomes and lysosomes, increasing the degradation rate of long-lived proteins in cells. In contrast, JNK inhibitors could block the effect of palmitic acid. In addition, PKR (double-stranded RNA-activated protein kinase) and JNK activation are parallel, and palmitic acid-induced islet *β*-cell autophagy activation may be achieved through the PKR-JNK pathway. Komiya et al. [46] found elevated level of LC3-II, as well as the upregulation of endoplasmic reticulum stress markers BiP and CHOP and P-JNK in mouse MIN6 cells after palmitic acid stimulation. Admission of JNK inhibitor could alleviate the increased LC3-II expression to some extent. Thus, palmitic acid-induced endoplasmic reticulum stress upregulates islet *β*-cell autophagy via JNK signaling pathway.

## 5. Conclusions

Taken together, we identified several key circRNAs that may regulate the autophagy of islet *β*-cells by circRNA microarray and qRT-PCR. By analyzing the targets of circRNAs and their association with islet *β*-cell autophagy, we found new targets for the autophagy of islet *β*-cells in T2DM, thus providing new ideas for the treatment of T2DM.

## Figures and Tables

**Table 1:** Primers used for qRT-PCR.

Primers	Sequence (5′-3′)
CircRNA_011190-RT-F	AGGAAAATGACAACTATTACGGCA
CircRNA_011190-RT-R	TCCCGGGAGTATCTGGTCTG
CircRNA_014301-RT-F	AGAATGAGTCGCTCCTGCAC
CircRNA_014301-RT-R	AGTACCGCTTGGCATGTGAG
CircRNA_000793-RT-F	GCCCCGATTTCTTTTACCGC
CircRNA_000793-RT-R	TGGAGGTCTGACTGCAGGTT
CircRNA_31436-RT-F	TCACCAAGGACAAGGGAGGA
CircRNA_31436-RT-R	CAGGAGAGGGTCTGGAGGAT
CircRNA_008695-RT-F	AGGAGGACAATGAAGCGCC
CircRNA_008695-RT-R	TCCCCATTGACACACAAGCA
CircRNA_001731-RT-F	TGTCCTCACTGTGACTACGC
CircRNA_001731-RT-R	CTCCCGATGGTGTCTCTCCA
CircRNA_017447-RT-F	GGCTCATCTGGTCGACCTTC
CircRNA_017447-RT-R	CTCTCCCTATGCACACCACG
CircRNA_008565-RT-F	AGGAGAAGAGCGTTGCTTGG
CircRNA_008565-RT-R	TGTAAGAATCCTGGCGGCTC
CircRNA_011560-RT-F	AGGTGGCAGAATCAGGTGTG
CircRNA_011560-RT-R	TGTTGTAACTCCCCAGAGCG
CircRNA_24828-RT-F	ATCTGCAGCTACGAGTGTCG
CircRNA_24828-RT-R	ACCGGTGAGAGTTTCTCCCT
Rat GAPDH-RT-F	ACAGCAACAGGGTGGTGGAC
Rat GAPDH-RT-R	TTTGAGGGTGCAGCGAACTT

**Table 2:** Differentially expressed circRNAs.

Probe ID	Fold change	FC (abs)	Log fold change	Regulation	CircRNA
ASCRPR006675	20.2298115	20.2298115	4.338411	Up	rno_circRNA_000740
ASCRPR000684	18.8167126	18.8167126	4.2339427	Up	rno_circRNA_011190
ASCRPR008895	17.0502792	17.0502792	4.0917235	Up	rno_circRNA_014301
ASCRPR005315	16.8565809	16.8565809	4.07524	Up	rno_circRNA_000793
ASCRPR003230	14.679558	14.679558	3.8757366	Up	rno_circRNA_013859
ASCRPR010484	11.7624297	11.7624297	3.5561142	Up	rno_circRNA_013860
ASCRPR008969	11.6792345	11.6792345	3.5458738	Up	mmu_circRNA_31436
ASCRPR008579	10.7448224	10.7448224	3.4255697	Up	rno_circRNA_008695
ASCRPR003118	−25.3551909	25.3551909	−4.6642092	Down	rno_circRNA_001731
ASCRPR004270	−24.0287976	24.0287976	−4.5866926	Down	rno_circRNA_017447
ASCRPR010233	−21.9741798	21.9741798	−4.4577374	Down	rno_circRNA_008565
ASCRPR005498	−21.7321047	21.7321047	−4.441756	Down	rno_circRNA_011560
ASCRPR007554	−20.2903139	20.2903139	−4.3427193	Down	mmu_circRNA_24828
ASCRPR013192	−15.2108669	15.2108669	−3.9270305	Down	rno_circRNA_008424
ASCRPR012113	−14.8760697	14.8760697	−3.8949215	Down	rno_circRNA_007118
ASCRPR006011	−14.3920522	14.3920522	−3.8472004	Down	mmu_circRNA_39417

**Table 3:** Identification of the top 10 circRNAs associated with islet *β*-cell autophagy.

CircRNA-related genes	Score with autophagy in GeneCards	CircRNA
CSF1	1.82	mmu_circRNA_24828
MAPK1	8.23	rno_circRNA_008565
MAPK8 (JNK)	9.15	rno_circRNA_008565
MAPK9	4.18	mmu_circRNA_24828
MAPT	4.78	mmu_circRNA_24828
DAB2IP	1.18	mmu_circRNA_24828
HRK	0.79	mmu_circRNA_24828
RIPK1	5.61	rno_circRNA_008565
RASSF5	1.4	mmu_circRNA_24828
CLOCK	0.91	rno_circRNA_008565

## Data Availability

The data used to support the findings of this study are available from the corresponding author upon request.
